# Survey on patient safety climate in public hospitals in China

**DOI:** 10.1186/s12913-015-0710-x

**Published:** 2015-02-07

**Authors:** Ping Zhou, M Kate Bundorf, Jianjun Gu, Xiaoyan He, Di Xue

**Affiliations:** Key Laboratory of Health Technology Assessment (MOH); School of Public Health, Fu Dan University, Shanghai, 200032 P.R. China; Department of Health Research and Policy, Stanford University School of Medicine, Stanford, USA; Commission of Health and Family Planning of Pudong New Area, Shanghai, P.R. China

**Keywords:** Patient safety, Public hospital, Hospital quality, China, Employee perception

## Abstract

**Background:**

Patient safety climate has been recognized as a core determinant for improving safety in hospitals. Describing workforce perceptions of patient safety climate is an important part of safety climate management. This study aimed to describe staff’s perceptions of patient safety climate in public hospitals in Shanghai, China and to determine how perceptions of patient safety climate differ between different types of workers in the U.S. and China.

**Methods:**

Survey of employees of 6 secondary, general public hospitals in Shanghai conducted during 2013 using a modified version of the U.S. Patient Safety Climate in Health Care Organizations (PSCHO) tool. The percentage of “problematic responses” (PPRs) was used to measure safety climate, and the PPRs were compared among employees with different job types, using *χ*^2^ tests and multivariate regression models.

**Results:**

Perceptions of patient safety climate were relatively positive among hospital employees and similar to those of employees in U.S. hospitals along most dimensions. For workers in Chinese hospitals, the scales of “fear of blame” and “fear of shame” had the highest PPRs, whereas in the United States the scale of “fear of shame” had among the lowest PPRs. As in the United States, hospital managers in China perceived a more positive patient safety climate overall than other types of personnel.

**Conclusions:**

“Fear of shame” and “fear of blame” may be important barriers to improvement of patient safety in Chinese hospitals. Research on the effect of patient safety climate on outcomes is necessary to implement effective polices to improve patient safety and quality outcomes in China.

**Electronic supplementary material:**

The online version of this article (doi:10.1186/s12913-015-0710-x) contains supplementary material, which is available to authorized users.

## Background

The 1999 report from the Institute of Medicine, To Err Is Human, brought greater attention to the problem of patient safety in the developed countries [1], arguably launching the modern patient safety movement [2]. The report highlighted the role of an organization’s safety culture as a determinant of risks to patients care. Developing a culture of safety has been a core element of many efforts and a critical determinant of the success of activities intended to improve safety [3].

Patient safety culture can refer to “the product of individual and group values, attitudes, competencies and patterns of behavior that determine the commitment to, and the style and proficiency of, an organization’s health and safety programs” [4]. The safety culture of an organization can motivate workers to engage in safe behaviors and facilitate the translation of these behaviors into daily practice, and can also influence the ability of staff to raise concerns regarding safety and the ability of managers to respond to those concerns [3,5]. Safety culture reflects the deeper and less readily accessible core values and assumptions of the organization regarding safety and human resources [6]. Safety climate, in contrast, is commonly defined as, “surface features of the safety culture from attitudes and perceptions of individuals at a given point in time” or “the measurable components of safety culture” [4,7,8]. Because safety culture is difficult to measure, employee surveys of patient safety climate often serve as a proxy for patient safety culture in studies of the effects of culture on organizational outcomes [9]. Studies have found that patient safety climate is associated with positive outcomes such as greater error reporting by physicians [10], lower rates of adverse events [11,12], lower mortality rates [13], and lower rates of readmission [14].

Patient safety has taken on particular importance in China due to the deteriorating relationship between physicians and patients. The delivery of unsafe medical care is one of the reasons for the trend toward increasing conflict. In response, the Chinese government and hospitals have carried out a series of initiatives to prevent medical errors, including encouraging the development of safety culture [15-18]. As clinicians and policy makers place more emphasis on patient safety, assessing patient safety climate has taken on greater importance.

While most of the research on patient safety climate has focused on the U.S., researchers have documented important differences between the U.S. and other countries. For example, administering the same survey to hospital workers in three different countries and regions, Fujita et al. (2013) documented differing perceptions of patient safety climate along particular dimensions across the three countries [19]. Nie found similar results comparing instruments fielded in the U.S. and China [20].

Researchers have also documented that survey instruments developed and calibrated for workers in U.S. hospitals often do not perform as well when administered in other countries [18]. In particular, researchers administering the Hospital Survey on Patient Safety Culture (HSPSC), developed by the U.S. Agency for Healthcare Research and Quality (AHRQ), outside the U.S. often identify different underlying constructs and generally document lower internal consistency reliability [18-20]. As a result, Zhu et al. (2014) developed a new instrument specific to the Chinese hospital context [18].

Most of this work, however, is based on the AHRQ instrument. In this paper, in contrast, we base our analysis on an alternative instrument, the Patient Safety Climate in Healthcare Organizations (PSCHO) tool, which we believe better captures certain underlying characteristics of Chinese culture that could be important determinants of patient safety climate. In particular, the instrument uses separate scales to measure of “fear of blame” and “fear of shame”, recognizing that they may operate differently and that the different scales may be more problematic among different types of employees [20,21]. “Fear of blame” and “fear of shame” affect patient safety by reducing the willingness of workers to share experiences and learn from others. We anticipated that these items would be particularly relevant for workers in Chinese hospital and thus chose the instrument that could better capture these separate elements. Theory of high-reliability organizations also suggests that these factors are potentially important barriers to implementing interventions to improve patient safety [22].

Our study focuses on general, secondary public hospitals in Shanghai. Shanghai is one of the biggest urban cities in China with a population of 23.80 million in 2013, and improving medical care quality and patient safety has been a key objective of reforms of the public hospitals. The Shanghai government has facilitated the use of clinical pathways, disease management programs, and computer-assisted quality and safety programs and has linked government subsidies to public hospitals to the quality and safety of their medical care. Understanding patient safety climate may help policy makers and hospital leaders better understand how to design these interventions to facilitate these types of reforms. In this paper, we report the results of fielding a modified version of the PSCHO in hospitals in Shanghai China. Using this instrument, we assess the status of patient safety climate in hospitals in China and compare the results to those reported from a study of U.S. hospitals.

## Methods

### Data source

We studied hospitals in Pudong new area of Shanghai, the largest district in Shanghai with both urban and rural areas, covering a population of about 5.26 million (22% of the total population in Shanghai) and an area of 1210.41 km^2^ (19% of the total area in Shanghai) [23]. We surveyed each of the six secondary general public hospitals in Pudong new area, four in the northern urban area and two in the southern rural area. In each hospital, we randomly sampled 50% of managers in administrative offices and clinical departments, 20% of non-management physicians, non-management nurses and others (including medical technicians and others with no management positions). For each category, we surveyed at least 30 people. Employees received a paper-based, voluntary and anonymous questionnaire. The survey was conducted during January and February of 2013.

### Weighting data

We generated weights for each job type within each hospital to reflect the original sampling frame accurately. Following Hartmann’s study [24], weights were calculated by multiplying the sampling weight by the non-response weight.

### Survey instrument

We chose a previously validated instrument, the Patient Safety Climate in Healthcare Organizations (PSCHO) tool, which was developed to study patient safety in U.S. hospitals [24-28]. The PSCHO, which is based on HRO theory [29], evaluates factors related to organizational, work-unit and individual/interpersonal contributions to patient safety climate and has been demonstrated to be a valid and reliable instrument [25]. The PSCHO contains 12 scales and 42 items [23]. There are three organizational scales (senior leadership, resources for safety, and facility characteristics), three workgroup scales (workgroup leadership, workgroup norms, and workgroup recognition), and six interpersonal scales (fear of shame, learning, fear of blame, psychological safety, problem responsiveness, and outcomes). The survey also asked employees to provide demographic information including gender, age, education, working years, and job type.

To adapt the tool to local culture, we adjusted some of the phrasing to better align the survey with culture-specific interpretations. We replaced the word “I” with “staff” or used passive sentences instead of original positive sentences. For example, “I have enough time to complete patient care tasks safely” in the PSCHO was modified as “Staff have enough time to complete patient care tasks safely”. There were two main reasons for this modification. First, we believe it is likely that Chinese respondents would be particularly sensitive to the use of the word “I” in this context, causing them to be unwilling to respond the survey or to respond inaccurately. Second, we believe this phrasing more accurately measures collective beliefs and attitudes about an employee’s work unit and hospital, and thus reflects organizational feature of safety climate. Although a 5-point, neutral mid-point Likert scale was used in the PSCHO, we used a 7-point, neutral mid-point Likert scale (“strongly disagree” = 1 to “strongly agree” = 7) with no “not applicable” option for each safety climate item in order to capture severe problems in patient safety climate (see Additional file 1).

### Administration of survey

Survey questionnaires were delivered to each hospital by members of our research group and were distributed within hospitals by each site’s project coordinator. Participation was voluntary and all responses were anonymous. Each survey questionnaire contained a cover letter that introduced the survey and informed respondents of their rights as research participants. The coordinator collected the completed questionnaires from each department and delivered them to the research team.

### Data analysis

Following analyses based on the PSCHO [24-28], we used the percentage of “problematic responses′ (PPR) to measure patient safety climate. A rating below 4 for a positive statement or above 4 for a negative statement was identified as a problematic response. A rating of 4 was identified as a neutral response. Weighting each survey item equally, we calculated the PPRs overall, for each scale and for item and calculated the PPRs by job type [24-28]. Because a neutral mid-point response (i.e., neither agree nor disagree) to an item could also be characterized as problematic with respect to safety climate [25], we also calculated the percentage of problematic and neutral responses for each measure.

After tabulating the responses for each item, we assessed the psychometric properties of the modified edition of the PSCHO instrument. First, we assessed the internal reliability for each scale based on the full set of items we included in the survey using Cronbach’s alpha. For scales that did not meet an acceptable threshold for Cronbach’s alpha (> = 0.7), we analyzed the correlation of the items within each scale and identified and dropped items that had relatively low correlation with the others. We then conducted a confirmatory factor analysis for the revised model and reanalyzed the internal reliability using Cronbach’s alpha. We then used the revised scales for the remainder of our analyses.

To determine whether perceptions of hospital safety climate varied by job type, we tested the difference of PPRs by job type using *χ*^2^ tests. We also used a multivariate regression model to control individual characteristics of employees (gender, education, and working years). The model also include hospital fixed effects to control for differences across hospitals. We estimated models for each scale. Before adopting multivariate regression model, we reversed the score for negative statements so that a higher score always indicated a more positive response. To test differences by job type, we included an indicator of whether the worker was a manager. For each respondent, the score for each multi-item dimension is the average across each of the items included in the dimension, and the overall safety climate score is the average across all the original items. Models were estimated using the weights discussed above.

The study was approved by the Institutional Review Board of School of Public Health, Fudan University (IRB#2012-11-0381).

## Results

### Respondent characteristics

1,272 of surveyed employees responded, a 75% response rate. The response rates for non-management physicians, non-management nurses, managers in administrative offices and clinical departments, and others were 81%, 85%, 77% and 46%, respectively. The majority of the study respondents were female (69%); 40% were above the age of 40; 14% had graduate degrees; 4% were managers in administrative offices; and 59% had worked in the hospital for 10 years or more (Table 1).

**Table 1:** **Demographic information of respondents**

**Items**	**N**	**%**	**Items**	**N**	**%**
Sex			Job types		
Male	391	30.84	managers	47	3.69
Female	877	69.16	physicians	505	39.70
Total	1268	100.00	nurses	534	41.98
Age (year)			others	186	14.62
<25	93	7.36	Total	1272	100.00
25-	172	13.62	Working years (years)		
30-	265	20.98	<10	512	40.93
35-	232	18.37	10-	422	33.73
40-	216	17.10	20-	248	19.82
45-	137	10.85	30-	69	5.52
50-	100	7.92	Total	1251	100.00
55-	48	3.80			
Total	1263	100.00			
Education					
PhD degree	29	2.30			
Master degree	142	11.28			
Bachelor degree	674	53.53			
College degree	368	29.23			
Others	46	3.65			
Total	1259	100.00			

When calculating the weights and analyzing the PPRs by job type, we classified as “managers” those who worked in administrative offices rather than in direct patient care (such as physician managers, nurse managers, and clinician managers). Front-line managers with clinical responsibilities, in contrast, were classified based on their clinical job groups in order to differentiate between employees who directly interacted with patients and those who did not.

### Psychometric properties of the PSCHO

93% of the original set of items in the modified PSCHO had correlations of 0.40 or higher with the other items within their scales. Ten scales had high internal consistency (Cronbach’s α coefficient ranging from 0.81 to 0.95), two scales of “workgroup leadership” and “outcome” had lower Cronbach’s α coefficient (0.26 and 0.37 respectively). In the scale of “workgroup leadership”, the item of “Whenever pressure builds up, management in the unit wants us to work faster, even if it means taking shortcuts that might negatively affect patient safety” had very low correlation with other items within the scale (less than 0.2). In the scale of “outcome”, the item of “I have never witnessed a coworker do something that appeared to me to be unsafe patient care” also had low correlation with other items within the scale (less than 0.3). To improve the psychometric properties of the scales, these two items were deleted. After deleting these items, the Cronbach’s α coefficients for the scales of “workgroup leadership” and “outcome” were 0.90 and 0.87, respectively.

After deleting the two items, we conducted a confirmatory factor analysis of the revised scales. The Standardized Root Mean Square Residual (SRMR) and the root mean square error of approximation (RMSEA) of the PSCHO were 0.069 and 0.071 respectively. Bentler’s comparative fit index was 0.92. The Bentler & Bonett’s normed fit index and Non-normed fit index were both above 0.9. But the Goodness of fit index (GFI), the adjusted GFI (AGFI) of the modified PSCHO were 0.80 and 0.77 respectively, slightly lower than the criterion of these two index (GFI > = 0.85, AGFI > = 0.80). The values of most indices were in the range indicative of an acceptable fit of the model to the data [30,31].

### Problematic response

The overall average of problematic responses across six hospitals was 15% (Table 2). The scales of “problem responsiveness” (4%), “workgroup norms” (4%) and “senior leadership” (5%) had lower rates of problematic responses and the scales of “fear of blame” (79%), “fear of shame” (41%) and “outcomes” (36%) had higher rates of problematic responses. The scales with lowest and highest rates of problematic responses were the same when considering both problematic and neutral responses.

**Table 2:** **Problematic responses** ^†^

**Scales and text of item (Cronbach’s α Coefficient)**	**% Problematic**	**% Problematic + % Neutral**
Senior leadership (0.95)	5.55	14.99
Good communication flow exists up and down the chain of command regarding patient safety issues	8.83	15.95
Senior management supports a climate that promotes patient safety	4.71	11.01
Senior management has a clear planning and actions to deal with the risks that associated with patient care	4.71	12.43
Senior management uses proper ways to deal with the mistakes that actually occur in this facility	4.74	12.74
Senior management considers patient safety when program changes are discussed	4.50	12.56
Patient safety decisions are made by people regardless of rank or hierarchy	5.90	15.18
Resources for safety (0.95)	8.91	21.66
Staff is provided with adequate resources (personnel, budget, and equipment) to provide safe patient care	8.69	19.25
Staff has enough time to complete patient care tasks safely	8.66	20.51
Staff has received sufficient training to enable them to address patient safety problems	9.40	19.64
This facility devotes sufficient resources to follow-up on identified safety problems	8.89	21.41
Facility characteristics (0.89)	7.06	17.69
Compared with other facilities in the area, this facility cares more about the equipment safety	9.06	18.05
Overall the level of patient safety at this facility is improving	5.06	12.78
Workgroup leadership (0.26)^‡^	23.61	32.73
Management in the unit helps staff overcome problems	6.80	15.41
Management puts safety at importance	5.29	12.37
Whenever pressure builds up, management in the unit wants us to work faster, even if it means taking shortcuts that might negatively affect patient safety	58.74	70.41
Workgroup norms (0.92)	4.13	13.15
My unit takes the time to identify and assess risks to ensure patient safety	3.67	10.44
My unit has risk management to ensure patient safety	3.62	11.06
We have learned how to do our job better by learning about mistakes	2.51	9.18
There is significant peer pressure to discourage unsafe patient care	8.07	17.77
Anyone found to violate standards or safety rules is corrected	2.74	9.74
Deliberate violations of standard operating procedures are rare	4.16	10.71
Workgroup recognition (0.90)	6.51	16.76
Taking quick action to identify a serious mistake is rewarded	6.92	15.26
Individual safety achievement is recognized through rewards	7.74	16.41
Teamwork is encouraged in order to improve patient safety in medical care	4.88	10.94
Fear of shame (0.95)	41.16	51.94
Asking for help is a sign of incompetence	42.10	51.34
People will not tell others about a mistake that has significant consequences and if nobody notices the mistake	39.82	50.11
Telling others about the mistakes is embarrassing	41.56	52.55
Learning (0.81)	12.76	23.07
Mistakes have led to positive changes in the unit	19.21	28.97
Personal performance is evaluated against defined safety standards	10.48	20.29
Patient safety problems and errors are communicated to the right people so that the problem can be corrected	8.58	17.00
Fear of blame (0.82)	78.53	88.37
If a person makes a mistake and is found, he will be disciplined.	76.14	85.63
Clinicians who make serious mistakes are usually punished	80.91	89.71
Psychological safety(0.95)	7.81	20.08
Staff can feel comfortable questioning the actions of those with more authority when patient safety is at risk	9.37	20.32
Staff can freely voice their opinions on patient safety.	7.72	19.04
Staff can freely identify events that may negatively affect patient safety	7.04	17.81
Staff can freely report patient safety incidents to the relevant administrative department in hospital.	7.10	19.57
Problem responsiveness(0.95)	3.84	12.32
Patient safety concerns usually results in the problem being addressed	3.74	11.28
We identify and fix safety problems timely	3.51	10.08
There is appropriate follow-up when patient safety issues are communicated	4.11	10.93
We will analyze the accidents or unexpected events timely	3.99	10.14
Outcomes (0.37)^△^	34.04	41.77
In the last year, I have witnessed a coworker do something that appeared to me to be unsafe for the patient	38.54	45.67
I have never witnessed a coworker do something that appeared to me to be unsafe patient care	29.24	38.33
I have done something that was not safe for the patient	34.33	41.10
Overall average (0.959)^#^	15.43	25.52

^†^Responses weighted for sampling and for non-response. ^‡^If the item of “Whenever pressure builds up, management in the unit wants us to work faster, even if it means taking shortcuts that might negatively affect patient safety” was deleted, the Cronbach’s α Coefficient, PPR and neutral percent rate of the scale of “workgroup leadership” would be 0.90, 6.04, and 9.48 respectively.

△If the item of “I have never witnessed a coworker do something that appeared to me to be unsafe patient care” was deleted, the Cronbach’s α Coefficient, PPR and neutral percent rate of the scale of “outcome” would be 0.87, 36.44, 7.63 respectively.

^#^If the two items of “whenever pressure builds up, management in the unit wants us to work faster, even if it means taking shortcuts that might negatively affect patient safety” and “I have never witnessed a coworker do something that appeared to me to be unsafe patient care” were deleted, the Cronbach’s α Coefficient, PPR and neutral percent rate of the overall safety climate would be 0.963, 14.04, 8.63 respectively.

In the scale of “fear of blame”, 76% of respondents indicated that a person would be disciplined if he was found making a mistake and 81% of respondents indicated that clinicians who made serious mistakes were usually punished. For the scales of “fear of shame”, 42% of respondents thought that asking for help was a sign of incompetence, 42% of respondents thought that telling others about mistakes was embarrassing, and 40% of respondents thought that people would not tell others about a mistake that had significant consequences if they thought that nobody would notice the mistake.

In the scale of “workgroup leadership”, 59% of the respondents reported that whenever pressure builds up, management in the unit wants them to work faster, even if it means taking shortcuts that might negatively affect patient safety.

For the scale of “outcomes”, 39% of respondents reported that they had witnessed a coworker do something that appeared to be unsafe patient care in the last year and 34% of respondents reported that they had done something that was unsafe for patient care (Table 2).

### Problematic response by job type

The PPRs varied by job type (Table 3). The overall PPR for patient safety was slightly lower for managers than for physicians, nurses and other types of workers. While managers reported relatively low PPRs for the scales of “resources for safety”, “facility characteristics”, “work group recognition”, “fear of shame”, and “fear of blame”, they reported relatively high rates of PPRs for the scales of “psychological safety”, and “outcomes”. The PPRs for physicians were particularly high relative to other types of workers for the scale of “workgroup leadership”, “fear of shame” and the PPRs for nurses were particularly high relative to other types of workers for the scale of “fear of blame”.

**Table 3:** **Safety climate in the hospitals by job-type, unadjusted** ^**†**^ **(PPR)**

	**Type of worker**					
**Scales**	**Physicians**	**Nurses**	**Managers**	**Others**	**All**	***χ*** ^**2**^
Senior leadership	6.11	4.07	3.27	7.91	5.57	228.09^*^
Resources for safety	8.91	8.80	0.96	10.17	8.91	76.81^***^
Facility characteristics	6.72	6.55	2.92	8.97	7.05	31.32^***^
Workgroup leadership	7.23	5.04	1.02	6.99	6.04	43.84^***^
Workgroup norms	4.50	2.86	4.47	5.91	4.13	148.49^***^
Workgroup recognition	7.51	5.17	2.22	8.18	6.51	73.81^***^
Fear of shame	45.91	40.05	36.12	37.39	41.16	94.73^***^
Learning	11.07	10.97	16.64	17.91	12.77	156.62^***^
Fear of blame	76.35	81.05	67.99	78.12	78.53	58.40^***^
Psychological safety	7.00	6.48	11.37	10.92	7.81	130.43^***^
Problem responsiveness	4.44	2.18	2.22	6.29	3.84	196.09^***^
Outcomes	40.26	38.66	42.50	26.36	36.44	173.41^***^
Overall	14.58	13.17	11.75	15.20	14.04	198.40^***^

^†^Calculated for all personnel and by job types for each scale and for safety climate overall, weighted for job type within each hospital.

*p < 0.05, ***p < 0.001.

In Table 4, we present the results of multivariate regression models which test more directly for differences between managers and non-managers and also control for individual (gender, education, and working years) and hospital characteristics which may influence responses. We find that managers reported more favorable scores than other types of workers for most scales except for “fear of shame”, “learning”, “fear of blame”, and “outcomes”. They also had more positive assessments of patient safety climate overall.

**Table 4:** **Analyses on the factors that influence perception of patient safety climate** ^**†**^

**Parameter**	**Senior leadership**		**Resources for safety**		**Facility characteristics**		**Workgroup leadership**		**Workgroup norms**	
	**β**	**t**	**β**	**t**	**β**	**t**	**β**	**t**	**β**	**t**
Intercept	5.78	61.76^***^	5.43	52.95^***^	5.64	55.99^***^	5.76	56.88^***^	6.03	72.38^***^
Hospital *(ref Hospital 6)* ^‡^										
Hospital1	−0.05	−0.43	−0.15	−1.27	−0.04	−0.31	−0.08	−0.71	−0.10	−1.02
Hospital2	0.13	1.20	−0.17	−1.38	−0.33	−2.82^**^	−0.01	−0.04	−0.15	−1.58
Hospital3	0.17	1.40	−0.04	−0.29	−0.09	−0.65	0.14	1.06	−0.12	−1.13
Hospital4	0.59	4.47^***^	0.67	4.60^***^	0.68	4.78^***^	0.63	4.36^***^	0.37	3.17^**^
Hospital5	−0.24	−2.41^*^	−0.08	−0.71	−0.21	−1.91	−0.21	−1.95	−0.47	−5.23^***^
Male^#^	−0.19	−2.40^*^	0.02	0.18	−0.07	−0.88	−0.20	−2.34^*^	−0.26	−3.68^***^
PhD or Master degree^#^	−0.17	−1.53	−0.12	−1.00	−0.20	−1.72	−0.12	−1.00	−0.07	−0.74
Manager^#^	0.30	3.18^**^	0.25	2.38^*^	0.28	2.78^**^	0.40	3.90^***^	0.25	2.96^**^
Working years	−0.01	−1.22	−0.01	−1.86	−0.005	−1.08	−0.01	−1.43	−0.003	−0.72
F	8.39^***^		5.99^***^		8.30^***^		8.05^***^		10.83^***^	
Adjust R^2^	0.05		0.04		0.05		0.05		0.07	
N	1168		1169		1170		1170		1156	
**Parameter**	**Workgroup recognition**		**Fear of shame**		**Learning**		**Fear of blame**		**Psychological safety**	
	**β**	**t**	**β**	**t**	**β**	**t**	**β**	**t**	**β**	**t**
Intercept	5.66	56.45^***^	4.41	25.94^***^	5.37	50.15^***^	2.73	23.28^***^	5.64	54.66^***^
Hospital *(ref Hospital 6)* ^‡^										
Hospital1	0.02	0.20	0.12	0.58	−0.08	−0.61	0.19	1.42	−0.35	−2.88^**^
Hospital2	−0.12	−1.04	0.89	4.48^***^	−0.24	−1.89	0.21	1.50	−0.22	−1.79
Hospital3	0.06	0.45	0.26	1.15	0.03	0.19	−0.33	−2.11^*^	−0.04	−0.31
Hospital4	0.64	4.52^***^	0.39	1.61	0.42	2.76^**^	−0.08	−0.47	0.39	2.65^**^
Hospital5	−0.23	−2.11^*^	0.05	0.26	−0.22	−1.88	0.16	1.28	−0.22	−1.99^*^
Male^#^	−0.27	−3.19^**^	−0.57	−4.05^***^	−0.03	−0.30	−0.05	−0.47	−0.24	−2.76^**^
PhD or Master degree^#^	−0.16	−1.38	−0.08	−0.41	0.03	0.22	0.02	0.17	−0.11	−0.93
Manager^#^	0.33	3.29^**^	0.10	0.57	0.10	0.93	0.13	1.08	0.34	3.26^***^
Working years	−0.0002	−0.04	0.001	0.15	−0.002	−0.32	−0.01	−2.54	−0.001	−0.28
F	8.91^***^		5.07^***^		3.08^**^		2.59^**^		6.34^***^	
Adjust R^2^	0.06		0.03		0.02		0.01		0.04	
N	1156		1154		1139		1155		1151	
**Parameter**	**Problem responsiveness**				**Outcomes**			**Overall**		
	**β**		**t**		**β**	**t**		**β**	**t**	
Intercept	5.96		66.34^***^		5.01	28.60^***^		5.45	80.28^***^	
Hospital *(ref Hospital 6)* ^‡^										
Hospital1	0.00		−0.01		0.09	0.45		−0.06	−0.77	
Hospital2	0.02		0.19		0.18	0.85		0.03	0.42	
Hospital3	0.06		0.54		−0.24	−1.02		0.01	0.11	
Hospital4	0.39		3.07^**^		−0.29	−1.17		0.46	4.77^***^	
Hospital5	−0.34		−3.50^***^		0.16	0.87		−0.19	−2.54^*^	
Male^#^	−0.33		−4.40^***^		−0.63	−4.31^***^		−0.22	−3.81^***^	
PhD or Master degree^#^	−0.18		−1.71		0.23	1.13		−0.09	−1.08	
Manager^#^	0.32		3.51^***^		−0.12	−0.65		0.20	2.86^**^	
Working years	−0.002		−0.38		−0.002	−0.29		−0.003	−1.01	
F	9.77^***^				3.17^***^			9.27^***^		
Adjust R^2^	0.06				0.02			0.06		
N	1152				1130			1102		

^†^Multivariable regression models; ^‡^hospital dummy variables; ^#^1: yes, 0: no; *P < 0.05, **P < 0.01,***P < 0.001.

## Discussion

The objective of our study was to describe staff’s perceptions of patient safety climate in public hospitals in Shanghai, China and to determine how perceptions of patient safety climate differ between hospital workers in the U.S. and China.

### Overall patient safety climate of hospitals

Employees of the secondary general public hospitals of Pudong new area reported similar levels of problematic responses overall (15.4%) as employees in U.S. hospitals (15.9%) based on a survey of 69 U.S. hospitals [32]. The variance, however, across scales is much greater in the secondary general public hospitals of Pudong new area (3.8% ~ 78.5%) than in U.S. hospitals (4.8% ~ 36.4%). Rates of problematic responses were much higher in the domains of “fear of shame” and “fear of blame” among employees in Chinese public hospitals.

### Greater prevalence of “fear of shame” and “fear of blame”

Employees in the secondary general public hospitals in Shanghai are much more likely to report fear of shame (41.2%) than those in in the U.S. hospitals (4.8%). The discrepancy may be attributed to cultural differences between workers in the U.S. and China. Previous studies found that Chinese society tends to be more collective than the Western society, which is called “familial collectivism” [33,34]. People admitting mistakes do not only embarrass themselves but also embarrass the team. Thus, workers may be reluctant to admit to making mistakes in China. We found that “fear of shame” is the highest among physicians. Many Chinese doctors disagree strongly with the statement, “human error is inevitable”, suggesting that physicians in China may believe that to be a professional they must be infallible [31]. Thus, admitting a mistake is viewed as a sign of incompetence. This is consistent with “fear of shame” being particularly high among physicians.

The study also revealed that employees in the secondary general public hospitals in Shanghai are more likely than their American counterparts to report fear of blame (78.5% versus 32.2%). These results may be driven by a punitive work environment in healthcare in China. All types of workers in hospitals in China may face a punitive work environment in their interactions either with managers, other co-workers and/or patients. This is reinforced by the studies of nurses’ perceptions of safety culture report that nurses believe they will be punished for making an error and are afraid of reporting an error [35]. In our study, nurses exhibited the highest PPR for the scale of “fear of blame” (81%).

The possibility that patients may initiate complaint procedures and apply for compensation for adverse events or healthcare errors may also generate reluctance among health care workers to report adverse events. Relationships between patients and providers are often acrimonious, making patients likely to report complaints and creating concern among providers over making information about mistakes public.

In contrast, employees in Chinese hospitals reported higher levels of psychological safety than those in U.S. hospitals. In particular, the PPR for “staff can feel comfortable questioning the actions of those with more authority when patient safety is at risk” was particularly low (9% vs. 22%). Employees in Chinese hospitals appear to be more willing to report safety issues when they perceive that they are not to be blamed.

### Negative impact of heavy workloads on patient safety

Heavy workloads of employees in public general hospitals in China are likely an important factor that negatively impacts patient safety. Physicians in China make decisions on diagnosis and treatment in 6.37 minutes on average during an outpatient visit [36]. In our study, about 59% of the physicians, nurses, managers and other employees believed that their unit management wanted them work faster, even if it might negatively affect patient safety. The environmental factors conducive to promoting patient safety identified by the IOM appear to be lacking in Chinese public hospitals [1].

### More positive perception from managers

Like previous studies [24,25,28,37], our study also showed that managers perceived the safety climate more favorably than other types of workers. One possible explanation is that, in China, patient safety efforts are driven mainly by hospital managers. In response to patient demands for higher quality and safer care, hospital managers have implemented a variety of interventions directed at quality and patient safety. These efforts may have made hospital managers in Shanghai respond more positively to the questions on patient safety climate. It is also possible, however, that these differences are driven by the extent to which the different types of workers are directly exposed to clinical care. In our study, managers were restricted to those with administrative rather than clinical responsibilities. Thus, they may have different perceptions of patient safety than frontline workers who interact more directly with patients.

### Cross cultural differences in measuring patient safety climate

Our results are consistent with other research demonstrating that patient safety instruments developed for U.S. hospital workers often do not perform as well when administered in other countries. While the PSCHO performed well overall on the sample of public hospitals in China, in its original form, internal consistency was low for two scales: workgroup leadership and outcomes. As a result, we modified these scales.

However, we also emphasize that the PSCHO captured important elements of patient safety climate in public hospitals in China that were not measured by the HSPSC, which has been more widely used in cross-cultural studies. In particular, the HSPSC does not include the items of “fear of shame” which was important aspects of patient safety climate and has slightly different ways of asking about “fear of blame”. We found these scales were particularly problematic for workers in hospitals in China.

### Limitations

The key limitation of our study was that the study sample was restricted to a single area of Shanghai. Thus the results cannot necessarily be generalized to other areas or regions of China. However, we note that, relative to studies that sampled hospitals more broadly across the county [17], our analysis was designed to generate representative samples within organizations. Thus, not only does our analysis include all the general hospitals of a particular district but also is representative of workers within the hospitals. Indeed, our results suggest that these types of analyses should be replicated in other areas of China to determine if other hospitals face similar challenges in worker perceptions of patient safety climate. Another issue is the difference in timing of the Chinese and American surveys. Our survey was conducted in early 2013 and we compared the findings to a U.S. survey conducted in 2004. Patient safety climate may have changed over time in the U.S. hospitals. We also note that we defined managers differently in our survey. In our survey, we defined managers as non-clinical workers with positions in administrative offices. In other surveys, managers are defined as department head or above including clinical department directors. The results comparing managers and non-managers are similar despite this difference.

## Conclusion

With the reform of public hospitals in China, many efforts have been made to improve patient safety in public hospitals. The surveyed public hospitals of Pudong new area showed the same level of overall safety climate as those in the United States, but point to two potential areas of concern. First, hospital workloads may represent a barrier to providing safer patient care. In addition, fear of blame and fear of shame among hospital workers may also restrict the ability of managers to implement effective patient safety programs.

### Notes

We calculate the response rate assuming that hospitals distributed the survey questionnaires randomly to hospital employees according to study design.

## Additional file

Additional file 1:
**Comparison of the Items in Hartmann's Study and in Our Study.**

## References

[1] Institute of Medicine. To Err is Human: Building a Safer Health System.[Institution of Medicine web site]. November 1, 1999. Available at. http://www.iom.edu/Reports/1999/To-Err-is-Human-Building-A-Safer-Health-System.aspx. Accessed on Oct 30, 2013.

[2] Wachter B.. Is the Patient Safety Movement in Danger of Flickering Out? [Wachter’s World web site]. February 18, 2013. Available at. http://community.the-hospitalist.org/2013/02/18/is-the-patient-safety-movement-in-danger-of-flickering-out/. Accessed on Dec 30, 2013.

[3] Weaver SJ, Lubomksi LH, Wilson RF, Pfoh ER, Martinez KA, Dy SM (2013). Promoting a culture of safety as a patient safety strategy: a systematic review. Ann Intern Med.

[4] Halligan M, Zecevic A (2011). Safety culture in healthcare: a review of concepts, dimensions, measures and progress. BMJ Qual Saf..

[5] Pronovost PJ, Miller MR, Wachter RM (2006). Tracking progress in patient safety: an elusive target. JAMA.

[6] Mearns KJ, Flin R (1999). Assessing the state of organization safety: culture or climate?. Current Psychology.

[7] Gaba DM, Singer SJ, Sinaiko AD, Bowen JD, Ciavarelli AP (2003). Differences in safety climate between hospital personnel and naval aviators. Hum Factors..

[8] Singer SJ, Vogus TJ (2013). Safety climate research: taking stock and looking forward. BMJ Qual Saf.

[9] Colla JB, Bracken AC, Kinney LM, Weeks WB (2005). Measuring patient safety climate: a review of surveys. Qual Saf Health Care..

[10] Braithwaite J, Westbrook MT, Travaglia JF, Hughes C (2010). Cultural and associated enablers of, and barriers to, adverse incident reporting. Qual Saf Health Care.

[11] Singer SJ, Lin S, Falwell A, Baker L (2009). Relationship of safety climate and safety performance in Hospitals. Health Serv Res.

[12] Mardon RE, Khanna K, Sorra J, Dyer N, Famolaro T (2010). Exploring relationships between Hospital patient safety culture and adverse events. J Patient Saf.

[13] Estabrooks CA, Tourangeau AE, Humphrey CK, Hesketh KL, Giovannetti P, Thomson D (2002). Measuring the Hospital Practice Environment: a Canadian Context. Res Nurs Health.

[14] Hansen L, Williams M, Singer S (2010). Perceptions of hospital safety climate and incidence of readmission. Health Serv Res.

[15] Cheng YM, Liu Y, Liu YM (2012). Review on research and application of adverse events reporting system in China (In Chinese). Chin Hosp Manage..

[16] Ji CL, Chen CC, Liu DY (2008). The application of patient safety goals in clinical practice (In Chinese). Chin Health Qual Manage..

[17] Hu R (2012). WHO release of the Chinese version of the patient safety curriculum guide: multi-professional edition (In Chinese). Chin Community Physician..

[18] Zhu J, Li L, Zhao H, Han G, Wu AW, Weingart SN (2014). Development of a patient safety climate survey for Chinese hospitals: cross-national adaptation and psychometric evaluation. BMJ Qual Saf.

[19] Fujita S, Seto K, Ito S, Wu Y, Huang CC, Hasegawa T (2013). The characteristics of patient safety culture in Japan, Taiwan and the United States. BMC Health Serv Res..

[20] Nie Y, Mao X, Cui H, He S, Li J, Zhang M (2013). Hospital survey on patient safety culture in China. BMC Health Serv Res..

[21] Chen IC, Li HH (2010). Measuring patient safety culture in Taiwan using the Hospital Survey on Patient Safety Culture (HSOPSC). BMC Health Serv Res..

[22] Weick KE, Sutcliffe KM (2001). Managing the Unexpected.

[23] Shanghai Municipal Bureau of Statistics and Shanghai Survey Office of National Bureau of Statistics of China. Shanghai Statistical yearbook. Beijing: China Statistics Press; 2013.

[24] Hartmann WC, Rosen KA, Meterko M, Shokeen P, Zhao S, Singer S (2008). An overview of patient safety climate in the VA. Health Serv Res.

[25] Singer SJ, Gaba DM, Geppert JJ, Sinaiko AD, Howard SK, Park KC (2003). The culture of safety: results of an organization-wide survey in 15 California Hospitals. Qual Saf Health Care.

[26] Singer S, Meterko M, Baker LC, Gaba D, Falwell A, Rosen A (2007). Workforce perceptions of hospital safety culture: development and validation of the patient safety climate in Healthcare Organizations Survey. Health Serv Res.

[27] Singer SJ (2007). Safety climate in US Hospitals, its measurement, variation, and relationship to organizational safety performance.

[28] Singer SJ, Falwell A, Gaba DM, Baker LC (2008). Patient safety climate in US Hospitals variation by management level. Med Care.

[29] Gaba DM, Howard SK, Jump B (1994). Production pressure in the work environment: California Anesthesiologists’ attitudes and experiences. Anesthesiology.

[30] Bentler PM, Bonett DG (1980). Significance tests and goodness of fit in the analysis of covariance structures. Psychol Bull.

[31] Hu L, Bentler PM (1999). Cutoff criteria for fit indexes in covariance structure analysis: conventional criteria versus new alternatives. Struct Equation Model.

[32] Singer SJ, Hartmann CW, Hanchate A, Zhao S, Meterko M, Shokeen P (2009). Comparing safety climate between two populations of hospitals in the United States. Health Serv Res.

[33] Trandis HC (1988). Cross-cultural studies of personality, attitudes and cognition.

[34] Yang KS, Yeh MH, Yang KS, Hwang KK, Yang CF (2005). Familism and pan-familism. Chinese indigenized psychology.

[35] Liu Y, Kalisch BJ, Zhang L, Xu J (2008). Perception of safety culture by nurses in Hospitals in China. J Nurs Care Qual.

[36] Sun G, Zhang XP (2010). Quantitative analysis on treatment duration per physician visit for outpatients in eastern, central and western areas of China. Chin Health Serv Manage.

[37] Pronovost PJ, Weast B, Holzmueller CG, Rosenstein BJ, Kidwell RP, Haller KB (2003). Evaluation of the culture of safety: survey of clinicians and managers in an Academic Medical Center. Qual Saf Health Care.

