# Supplementary material for: Survey on patient safety climate in public hospitals in China
Source: BMC Health Serv Res. 2015 Feb 7;15:53. doi: 10.1186/s12913-015-0710-x (PMC4326494; doi:10.1186/s12913-015-0710-x)
Supplement: Additional file 1: — Comparison of the Items in Hartmann's Study and in Our Study. [file 12913_2015_710_MOESM1_ESM.docx]

**Appendix 1: Comparison of the Items in Hartmann's Study and in Our Study**

| **Items in Hartmann's Study^†^** | |  | **Modified Items in Our Study ^‡^**  **(English Version)** | |  | **Modified Items in Our Study**  **(Chinese Version)** | |
| --- | --- | --- | --- | --- | --- | --- | --- |
| Senior leadership | |  | Senior leadership | |  | 上级管理承诺 | |
|  | Good communication flow exists up and down the chain of command regarding patient safety issues |  |  | Good communication flow exists up and down the chain of command regarding patient safety issues |  |  | 可以自由地谈论与患者安全相关的话题 |
|  | Senior management supports a climate that promotes patient safety |  |  | Senior management supports a climate that promotes patient safety |  |  | 上级主管部门支持与促进患者安全 |
|  | Senior management has a clear picture of the risks associated with patient care. |  |  | Senior management has a clear planning and actions to deal with the risks that associated with patient care |  |  | 上级对患者安全风险有清晰的规划和处理方法 |
|  | Senior management has a good idea of the kinds of mistakes that actually occur in this facility |  |  | Senior management uses proper ways to deal with the mistakes that actually occur in this facility |  |  | 上级对错误有合适的处理方法 |
|  | Senior management considers patient safety when program changes are discussed |  |  | Senior management considers patient safety when program changes are discussed |  |  | 当更改工作程序或流程时上级会首先考虑患者的安全 |
|  | Patient safety decisions are made by the most qualified people regardless of rank or hierarchy |  |  | Patient safety decisions are made by people regardless of rank or hierarchy |  |  | 对患者安全问题作决定不被级别限制 |
| Resources for safety | |  | Resources for safety | |  | 组织安全资源 | |
|  | I am provided with adequate resources (personnel, budget, and equipment) to provide safe patient care |  |  | Staff is provided with adequate resources (personnel, budget, and equipment) to provide safe patient care |  |  | 有充足资源（人员、预算和设备）为患者提供安全的治疗/护理 |
|  | I have enough time to complete patient care tasks safely |  |  | Staff has enough time to complete patient care tasks safely |  |  | 有足够的时间为患者提供安全的治疗/护理 |
|  | I have received sufficient training to enable me to address patient safety problems |  |  | Staff has received sufficient training to enable them to address patient safety problems |  |  | 提供培训的机会以提高解决患者安全问题的能力 |
|  | This facility devotes sufficient resources to follow-up on identified safety problems |  |  | This facility devotes sufficient resources to follow-up on identified safety problems |  |  | 投入充足的资源与设施去解决患者的安全问题 |
| Facility characteristics | |  | Facility characteristics | |  | 设施便利性 | |
|  | Compared with other facilities in the area, this facility cares more about the quality of patient care it provides |  |  | Compared with other facilities in the area, this facility cares more about the equipment safety |  |  | 与其它地方的设备相比本医院的设备更加注重患者安全 |

Continuous: Appendix 1

| **Items in Hartmann's Study^†^** | |  | **Modified Items in Our Study ^‡^**  **(English Version)** | |  | **Modified Items in Our Study**  **(Chinese Version)** | |
| --- | --- | --- | --- | --- | --- | --- | --- |
|  | Overall the level of patient safety at this facility is improving |  |  | Overall the level of patient safety at this facility is improving |  |  | 总的来说，本医院内，患者使用的设施安全保证不断提高 |
| Workgroup leadership | |  | Workgroup leadership | |  | 科主任领导能力 | |
|  | Management in my unit helps me overcome problems that make it hard for me to provide safe patient care |  |  | Management in the unit helps staff overcome problems |  |  | 工作中遇到困难时，领导能提供帮助 |
|  | In my unit, management puts safety at the same level of importance as meeting the schedule and productivity |  |  | Management puts safety at importance |  |  | 行政部门重视患者安全问题 |
|  | Whenever pressure builds up, management in my unit wants us to work faster, even if it means taking shortcuts that might negatively affect patient safety |  |  | Whenever pressure builds up, management in the unit want us to work faster, even if it means taking shortcuts that might negatively affect patient safety^*^ |  |  | 当压力积聚时领导总是希望我们加快工作速度即使可能会不利于患者安全 |
| Workgroup norms | |  | Workgroup norms | |  | 工作部门风气 | |
|  | My unit takes the time to identify and assess risks to ensure patient safety |  |  | My unit takes the time to identify and assess risks to ensure patient safety |  |  | 确认和评估各种风险以保证患者安全 |
|  | My unit does a good job managing risks to ensure patient safety |  |  | My unit has risk management to ensure patient safety |  |  | 有风险管理措施以保证患者的安全 |
|  | I have learned how to do my own job better by learning about mistakes made by my coworkers |  |  | We have learned how to do our job better by learning about mistakes |  |  | 我们会从错误中学习如何更好地把工作做好 |
|  | In my unit, there is significant peer pressure to discourage unsafe patient care |  |  | There is significant peer pressure to discourage unsafe patient care |  |  | 来自同事的压力会显著阻止不利于患者安全的行为 |
|  | In my unit, anyone found to intentionally violate standards or safety rules is corrected |  |  | Anyone found to intentionally violate standards or safety rules is corrected |  |  | 发现有意违背医疗安全规范的行为都给予纠正 |
|  | Deliberate violations of standard operating procedures are rare in my unit |  |  | Deliberate violations of standard operating procedures are rare |  |  | 故意违反操作规程或标准的行为是罕见的 |
| Workgroup recognition | |  | Workgroup recognition | |  | 工作部门的重视程度 | |
|  | I am rewarded for taking quick action to identify a serious mistake |  |  | Taking quick action to identify a serious mistake is rewarded |  |  | 快速识别出不利于患者安全的重大错误的个人受到奖励 |
|  | My unit recognizes individual safety achievement through rewards |  |  | Individual safety achievement is recognized through rewards |  |  | 取得安全业绩的个人受到奖励 |

Continuous: Appendix 1

| **Items in Hartmann's Study^†^** | |  | **Modified Items in Our Study ^‡^**  **(English Version)** | |  | **Modified Items in Our Study**  **(Chinese Version)** | |
| --- | --- | --- | --- | --- | --- | --- | --- |
|  | My unit provides training on teamwork in order to improve patient care performance and safety |  |  | Teamwork is encouraged in order to improve patient safety in medical care |  |  | 提倡团队协助，以提升病人医疗、护理安全 |
| Fear of shame | |  | Fear of shame | |  | 害怕受羞辱 | |
|  | Asking for help is a sign of incompetence* |  |  | Asking for help is a sign of incompetence^*^ |  |  | 寻求帮助是无能力的表现 |
|  | If I make a mistake that has significant consequences and nobody notices, I do not tell anyone about it* |  |  | People will not tell others about a mistake that has significant consequences and if nobody notices the mistake^*^ |  |  | 严重的错误若没有被别人发现将不会告诉任何人 |
|  | Telling others about my mistakes is embarrassing* |  |  | Telling others about mistakes is embarrassing^*^ |  |  | 告诉别人犯了错误是件非常尴尬的事 |
| Learning | |  | Learning | |  | 差错的学习与分享 | |
|  | Mistakes have led to positive changes in my unit |  |  | Mistakes have led to positive changes in the unit |  |  | 错误导致单位的积极变化 |
|  | My performance is evaluated against defined safety standards |  |  | Personal performance is evaluated against defined safety standards |  |  | 依据界定的安全标准评估个人表现 |
|  | In my unit, patient safety problems and errors are communicated to the right people so that the problem can be corrected |  |  | Patient safety problems and errors are communicated to the right people so that the problem can be corrected |  |  | 有关患者的安全问题和错误传达给合适的人可以使问题得到纠正 |
| Fear of blame | |  | Fear of blame | |  | 害怕受责备 | |
|  | If people find out that I made a mistake, I will be disciplined |  |  | If a person makes a mistake and is found, he will be disciplined. ^*^ |  |  | 个人被发现犯了错误会受到纪律处分 |
|  | Clinicians who make serious mistakes are usually punished |  |  | Clinicians who make serious mistakes are usually punished.^*^ |  |  | 医生犯了严重错误通常会受处罚 |
| Psychological safety | |  | Psychological safety | |  | 心理安全 | |
|  | Staff feel comfortable questioning the actions of those with more authority when patient safety is at risk |  |  | Staff can feel comfortable questioning the actions of those with more authority when patient safety is at risk |  |  | 员工通常都能坦然向上级行政人员提出关于患者安全的疑问 |
|  |  |  |  | Staff can freely voice their opinions on patient safety |  |  | 员工通常能自由发表关于患者安全的意见 |
|  | Staff freely speak up if they see something that may negatively affect patient care |  |  | Staff can freely identify events that may negatively affect patient safety. |  |  | 员工通常能自由指出可能会对患者安全造成不利影响的事件 |
|  | I am comfortable reporting safety concerns without fear of being punished by management |  |  | Staff can freely report patient safety incidents to the relevant administrative department in hospital. |  |  | 员工通常会自由地报告患者安全的事件 |

Continuous: Appendix 1

| **Items in Hartmann's Study^†^** | |  | **Modified Items in Our Study ^‡^**  **(English Version)** | |  | **Modified Items in Our Study**  **(Chinese Version)** | |
| --- | --- | --- | --- | --- | --- | --- | --- |
| Problem responsiveness | |  | Problem responsiveness | |  | 患者安全问题的反应性 | |
|  | Bringing patient safety concerns to management’s attention usually results in the problem being addressed |  |  | Patient safety concerns usually results in the problem being addressed |  |  | 患者的安全隐患能够妥善得到解决 |
|  | In my unit, we identify and fix safety problems before an incident actually occurs |  |  | We identify and fix safety problems timely |  |  | 我们会及时处理安全问题 |
|  | When I take time to communicate about patient safety problems there is appropriate follow-up |  |  | There is appropriate follow-up when patient safety issues are communicated |  |  | 患者安全的话题能得到积极的响应 |
|  | Our process of accident incident investigation is effective at identifying root causes |  |  | We will analyze the accidents or unexpected events timely |  |  | 我们会对于意外事故或事件及时分析 |
| Outcomes | |  | Outcomes | |  | 造成的不良结果 | |
|  | In the last year, I have witnessed a coworker do something that appeared to me to be unsafe for the patient* |  |  | In the last year, I have witnessed a coworker do something that appeared to me to be unsafe for the patient^*^ |  |  | 去年看见同事做了不利于患者安全的事情 |
|  | I have never witnessed a coworker do something that appeared to me to be unsafe patient care |  |  | I have never witnessed a coworker do something that appeared to me to be unsafe patient care |  |  | 从来没有见到同事做不利于患者安全的事情 |
|  | In the last year, I have done something that was not safe for the patient* |  |  | I have done something that was not safe for the patient^*^ |  |  | 自己曾经做了对患者安全不利的事情 |

† Hartmann, W.C., K.A. Rosen, M. Meterko, P. Shokeen, S. Zhao, S. Singer, A. Falwell, and D.M. Gaba. 2008. “An Overview of Patient Safety Climate in the VA.” *Health Services Research* 43(4):1263-1284.

‡ The underlined words or sentences mean the modified items in our study.

* The sentence is negative.
